# Treatment and research lines for the patient with COVID-19. What do we have and where are we going?

**DOI:** 10.1590/S1677-5538.IBJU.2020.S118

**Published:** 2020-07-27

**Authors:** Carolina Gotera

**Affiliations:** 1 IIS-Fundación Jiménez Díaz - ISCIII-CIBERES Madrid Spain Servicio de Neumología, IIS-Fundación Jiménez Díaz - ISCIII-CIBERES, Madrid, Spain

**Keywords:** Primary Prevention, Quarantine, COVID-19 diagnostic testing [Supplementary Concept]

## Abstract

Coronavirus disease 2019 (COVID-19) caused by severe acute respiratory syndrome coronavirus 2 (SARS-CoV-2) represents the most significant global public health crisis of this generation. From the beginning of the pandemic, several publications and on-line resources about different treatment lines have been done, and development effort in response to the COVID-19 pandemic to investigate potential therapies is unprecedented. Unfortunately, until now, there is not enough evidence to recommend any specific anti-COVID19 treatment. Randomized clinical trials and high-quality evidence, even in the middle of a pandemic, are needed. We provide a review of the latest published literature on the therapeutic strategies and current investigational lines for SARS-CoV-2.

## INTRODUCTION

Since the first case reported by Coronavirus disease (COVID-19) in Wuhan (1) China at the end of 2019 (2) to April 27, 2020, there have been > 3 million cases around the World, being Spain the first country in Europe with more than 229,000 cases and 23,500 deaths (3). COVID-19 is caused by severe acute respiratory syndrome coronavirus 2 (SARS-CoV-2). Patients with the disease may have mild symptoms up to more severe in a few days, such as fever, dry cough, myalgia, fatigue, diarrhea, dyspnea, and pneumonia in X--ray. In patients with COVID-19 pneumonia, an immune-mediated “cytokine storm” with several pro-inflammatory agents (IL-6, IL-8, IL-1β, granulocyte-macrophage colony-stimulating factor, and reactive oxygen species) and chemokines (such as CCL2, CCL-5, IFNγ-induced protein 10 (IP-10), and CCL3) all contribute to the acute respiratory failure and acute respiratory distress syndrome (ARDS). Approximately 20 to 41% of hospitalized patients with pneumonia developed ARDS (1). Indeed, ARDS is the leading cause of death in patients infected with SARS-CoV or Middle East respiratory syndrome (MERS-CoV). In a postmortem assessment of COVID-19 patients with severe ARDS, specimens of infected lungs demonstrated bilateral diffuse alveolar damage with edema, pneumocyte desquamation, and hyaline membrane formation (4).

From the beginning of the pandemic, several publications and on-line resources about different treatments lines have been done. Nevertheless, there is insufficient evidence to support the safety or efficacy of the treatments used for COVID-19 (5).

Our aim is to review the therapeutic strategies and current investigational lines for SARS-CoV-2.

## EVIDENCE ACQUISITION

This article will review the current evidence regarding the major proposed treatments, or experimental for COVID-19. We conducted a literary, comprehensive English-language literature research for original and review articles using the PubMed and Clinical trials database until May 2020. We search for the following MeSH terms: “COVID-19”, “SARS-CoV-2”, “coronavirus”, “2019-nCoV”, “Treatment”, “Immunotherapy”, “monoclonal antibody”, “vaccine”, “interleukin”, “Immunomodulation”, “Cytokines”, “clinical trial”. We included papers containing information on patients or treatments being considered for and undergoing clinical trials.

## EVIDENCE SYNTHESIS

### Understanding SARS-CoV-2 virus:

The genome sequencing indicated that the SARS-CoV-2 is a RNA betacoronavirus (1). SARS-CoV-2 has five major protein regions for virus structure assembly and viral replication (6). The cellular entry of coronaviruses depends on the binding of the spike (S) protein to a specific cellular receptor and subsequent S protein priming by cellular proteases. (2). The spike is a transmembrane glycoprotein that plays a pivotal role in mediating viral infection through binding the host receptor (6). Similarly, to SARS-CoV, SARS-CoV-2 employs the angiotensin-converting enzyme 2 (ACE2) as a receptor for cellular entry (2). The binding affinity of the S protein and ACE2 was found to be a significant determinant of the SARS-CoV replication rate and disease severity (2). The viral entry also depends on a host type 2 transmembrane serine protease (TMPRSS2) that facilitates cell entry via the S protein (2, 7). Once inside the cell, viral polyproteins synthesized RNA via RNA polymerase, and they release the viral particles. (7). These viral mechanisms provide potential and promising drug targets, such as 3-chymotrypsinlike protease, papain-like protease, RNA-dependent RNA polymerase. Additional drug targets include viral entry and immune regulation pathways (7). Table-1 summarizes the mechanism of action of select treatments or adjunctive therapies for COVID-19.

**Table 1:** Summarizes the mechanism of action of select treatments or adjunctive therapies for COVID-19.

Drugs/Agents	Target	Doses (adults)	Adverse events	Observations
**Chloroquine and hidroxichloroquine**	Blockade of viral entry and immunomodulatory effects through inhibition of cytokine production (both share the same mechanism).	500 mg by mouth every 1224 hours for 5 or 7 days (500 mg of chloroquine phosphate [salt] = 300 mg chloroquine base).	Abdominal cramps, anorexia, diarrhea, nausea, vomiting, QTc prolongation, hemolysis in G6PD deficiency, hypoglycemia, retinal toxicity, neuropsychiatric, and central nervous system effects.	Creatinine clearance <10 mL/min administer 50% of the dose. Hepatic: No dose adjustments (use with caution).
**Lopinavir/ritonavir**	Inhibiting 3C-like protease (3CLpro).	400/100 mg by mouth every 12 hours for up to 5-10 days maximum (at the beginning of the symptoms, first 7-10 days).	Diarrhea, nausea, vomiting, hepatotoxicity, hypertriglyceridemia and hypercholesterolemia, anxiety, headache, myalgia, pancreatitis.	No kidney or hepatic dose adjustments recommended (use with caution in hepatic impairment).
**Remdesivir**	RNA polymerase inhibitor.	Loading dose 200 mg intravenous followed 100 mg intravenous of maintenance once daily from day 2 to 10.	The main side effect is hypotension due to infusion. Other possible adverse reactions are nausea, vomiting, diarrhea, constipation, and abdominal pain.	Not recommended if creatinine clearance <3Ο mL/min.
**Tocilizumab**	IL-6 inhibition-reduction in cytokine storm.	Dose adjustments by weight: - ≥ 75 kg 600 mg one dose. - < 75 kg 400 mg one dose. Second dose 8-12 hours after the first dose if inadequate response.	Increase in upper respiratory tract infections (including tuberculosis), nasopharyngitis, headache, hypertension, increased AST, infusion-related reactions. Hematologic effects, infections, hepatotoxicity, gastrointestinal perforations, hypersensitivity reactions.	No dose adjustments recommended in mild or moderate kidney impairment. No hepatic dose adjustments recommended (not studied). Caution in patients with neutropenia (<500 cells/μL) or thrombocytopenia (<50000/μL)
**Metilprednisolone**	Regulate a vast array of physiological processes, and synthetic derivatives of these molecules are widely used in the clinic for treating inflammatory disorders, autoimmune diseases.	40 to 80 mg / IV / day, without exceeding 2 mg / kg (maximum 5 days).	Most frequent Nausea, vomiting, heartburn, headache, dizziness, trouble sleeping, appetite changes, increased sweating, or acne may occur.	kidney or hepatic failure (caution).
**Favipiravir**	RNA polymerase inhibitor.	Doses vary based on indication; limited data available.	Hyperuricemia, diarrhea, elevated transaminases, reduction in neutrophil count.	No required kidney adjustment (limited data available). Dose adjustment in Child-Pugh C is recommended.
**Anakinra**	Antagonist of IL-1β. The inhibition of IL-1β reduces the cytokine storm caused by infection.	100 mg subcutaneous injection per day.	Diarrhea, fever or chills, headache, itching, pain, redness, swelling, tenderness or warmth on the skin, joint pain, muscle aches and pains, nausea or vomiting, runny nose or sneezing and sore throat.	Creatinine clearance <30 mL/min or terminal renal failure (dialysis included) administer 100% of de dose every other day. Severe hepatic failure (caution).

## PHARMACOLOGICAL INTERVENTIONS IN COVID-19: WHAT WE HAVE?

COVID-19 viral infection is a life-threatening disease. There is insufficient evidence to support the safety or efficacy of the drugs used in COVID-19. Also, even some of the treatments used are under the support of clinical trials or compassionate use. Below, we reviewed the published clinical experiences of some of the most promising repurposed drugs for COVID-19.

### Antimalarial: Are they useful and safe to treat COVID-19 patients?

Chloroquine and hydroxychloroquine are drugs used in the treatment of malaria, systemic lupus erythematosus (SLE) and rheumatoid arthritis (RA) (7). Also, it has been demonstrated to have an anti-SARS-CoV activity *in vitro* (8). Briefing news from China reported chloroquine was successfully used to treat a series of more than 100 COVID-19 cases improving lung imaging findings, promoting virus-free conversion, and reduced disease progression (7).

A review by Cortegiani et al. of six articles published in March 2020 on the efficacy and safety of chloroquine for COVID-19 treatment suggested there is sufficient preclinical evidence to justify clinical research on the topic and extrapolated the safety of chloroquine on its existing use in clinical practice for other indications (8). In an observational study by Gautret et al., 80 patients with confirmed COVID-19 were treated with hydroxychloroquine in combination with azithromycin for at least three days and, followed up for at least six days. The majority of patients (92%) in the study had a low degree of illness (National Early Warning Score (NEWS) 0 - 4). The authors reported that 81.3% of patients who had a favorable outcome were discharged and, 83% had a fall in nasopharyngeal viral load, testing negative on day seven (9) Despite its small sample size, hydroxychloroquine treatment is significantly associated with viral load reduction/disappearance in COVID-19 patients, and its effect is reinforced by azithromycin (9).

Also, studies of chloroquine prophylaxis in healthcare workers (NCT04303507) and hydroxychloroquine for post-exposure prophylaxis after high-risk exposures (NCT04308668) are planned or enrolling. However, all the commented studies have important limitations, including small sample size, limited long-term outcome follow-up, etc… (7).

A few data exist regarding the optimal dose to ensure safety and efficacy. However, the recommended treatment for hydroxychloroquine in COVID-19 disease is 400 mg twice daily for one day, followed by 200 mg twice daily for five or seven days. Further studies are needed to determine the adequate dose. These drugs are relatively well tolerated. However, studies have reported adverse events, such as QTc prolongation and potential arrhythmias, especially in combination with QT-interval prolonging medications such as azithromycin or fluoroquinolones (electrocardiography to evaluate prolonged QT is recommended). (7). Even United States President Trump publically advocated the use of hydroxychloroquine and azithromycin in COVID-19. (Following this event, news of chloroquine poisoning from inappropriate over the counter use of the medication, including a report of a fatality, have surfaced in the U.S. and Nigeria (10)). Borba et al. investigated high versus low dose chloroquine (NCT04323527) in 81 patients and found more patients in the high dose chloroquine arm presented with QTc >500 mms (25%) when compared to the lower dose arm. The mortality rate was 13.5% (95% CI 6.9 - 23%), and recruitment was halted early in this arm because of adverse events. (11). Other side effects reported are hypoglycemia, neuropsychiatric effects, retinopathy diarrhea, vomiting, abdominal pain, nausea, and rash or itch (12). The use of chloroquine and hydroxychloroquine in pregnancy is considered safe. (7).

There is lack of robust evidence to conclude about the effectiveness and safe of these drugs. However, ongoing clinical trials could determine the role of chloroquine and hydroxychloroquine in this disease.

### Antivirals: Yes or Not? that is the question

Lopinavir/ritonavir is an oral drug approved for human immunodeficiency virus (HIV) treatment and is perhaps one of the most studied drugs. There is no published data for lopinavir/ritonavir *in vitro* activity for SARS-CoV-2 (7). Recent reports from Cao et al. comparing the efficacy of lopinavir/ritonavir vs standard care in adults hospitalized with severe COVID-19 found that this drugs did not have time for clinical improvement different from those patients assigned to standard of care alone in the intention-to-treat population (median, 16 days vs. 16 days; hazard ratio for clinical improvement, 1.31; 95% confidence interval [CI], 0.95 to 1.80; P=0.09). Also, it did not reduce mortality, or diminish throat viral RNA detectability in patients with serious COVID-19 (19.2% vs 25.0%: absolute difference, −5.8% [95% CI, −17.3% to 5.7%]) (13).

The Wuhan University Quick Guide for the treatment of patients with COVID-19 infection makes a weak recommendation in favor of the use of oral lopinavir/ritonavir, clarifying that if the window for treatment is lost, it is not longer effective, this guideline does not recommend other antivirals (10). The administration at the beginning of the symptoms (first 7-10 days) appears to be important, delayed therapy initiation with lopinavir/ritonavir did not affect clinical outcomes (7). Current data suggest a limited role for lopinavir/ritonavir in COVID-19 treatment.

The most used and studied lopinavir/ritonavir dosing regimen for COVID-19 treatment is 400mg/100mg twice daily for up to 14 days (13). A randomized controlled trial (RCT) showed nearly 50% of patients experienced an adverse event, and 14% of recipients were unable to complete the full 14-day course of administration (13). The adverse effects included nausea and diarrhea, hepatotoxicity with elevated transaminases by combination therapy, or viral infection in approximately 20% to 30% of patients (10).

– Remdesivir formally known as GS-5734 (a nucleotide analog prodrug that inhibits viral RNA polymerases who has demonstrated activity against SARS-CoV-2 *in vitro*) has shown improvement in oxygen-support status in 68% of patients, mortality rate was 13% over a median follow-up of 18 days (14). The interpretation of the results of this study is limited. Clinical trials are ongoing to evaluate the safety and antiviral activity of Remdesivir in COVID-19 (NCT04292899, NCT04292730, NCT04257656, NCT04252664, NCT04280705).
– Ribavirin: Its activity against other corona-viruses makes it a candidate for COVID-19 treatment. However, its *in vitro* activity against SARS-CoV was limited and required high concentrations to inhibit viral replication and combination therapy (7). It can cause severe adverse events such as hemolytic anemia reported in more than 60% of patients in SARS trial with a high dose (7). Other adverse events reported diarrhea, nausea, stomatitis, and transaminase elevations (15). Ribavirin is also a known teratogen and contraindicated in pregnancy (7).
– Favipiravir (T-705 - the active agent inhibits the RNA polymerase, halting viral replication). In an open-label clinical trial involving two treatment arms in patients with SARS-CoV-2 (favipiravir and lopinavir/ritonavir), the favipiravir arm performed better than the reference arm in terms of disease progression and clearance values (16). High doses should be considered for COVID-19. A loading dose of 2400mg to 3000mg every 12 hours by two doses, followed by a maintenance dose (1200mg to 1800mg every 12 hours). In general, the drug is well-tolerated, although the adverse event profile for higher-dose regimens is limited (7). (NCT04346628 NCT04359615; NCT04336904; NCT04349241; NCT04358549; NCT04303299; NCT04310228; NCT04333589).
– Oseltamivir (a neuraminidase inhibitor approved for the treatment of influenza) has no documented *in vitro* activity against SARS-CoV2) and has no role in the management of COVID-19 once the disease has been excluded (7).
– Umifenovir (also known as Arbidol, unavailable in Spain) has a mechanism of action targeting the S protein/ACE2 interaction and inhibiting membrane fusion of the viral envelope. The current dose of 200mg orally every 8 hours for influenza is being studied for COVID-19 treatment (7). Several clinical trials are ongoing, on monotherapy (NCT04260594) or in combination (NCT04252885, NCT04273763, NCT04261907, NCT04286503, NCT04350684, NCT04323345, NCT04333589) (16).

### Monoclonal antibodies:

– **Tocilizumab** (IL-6 receptor antagonist) has been used in a small series of severe COVID-19 cases with success early reports (7). IL-6 levels increase significantly in patients with severe COVID-19 (16). A report of 21 patients with COVID-19 showed that tocilizumab treatment, 400mg, was associated with clinical improvement in 91% of patients, measured by improved respiratory function, and successful discharge (7). For patients with reduced efficacy of the first dose, additional treatment can be applied after 12 hours (the prescription is the same as before), with a maximum of two cumulative doses (16). Tocilizumab is included in several RCT in Europe and United States (NCT04356937 NCT04346355, NCT04345445, NCT04331795, NCT04317092, NCT04320615, NCT04361552, NCT04332094, NCT04320615).
– **Anakinra**: (antagonist of IL-1β) the inhibition of IL-1β reduces the cytokine storm caused by infection. Data from a phase 3 randomized controlled trial of Anakinra IL-1 blockade in sepsis with Macrophage Activation Syndrome (MAS) characteristics showed a significant improvement in the 28-day survival rate (65.4% Anakinra vs. 35.3% placebo), with HR of fatal outcome of 0.28 (0.11-0.71, p = 0.0071), with no increased adverse events. Thus, anakinra could have a potential use to reduce systemic inflammation and lung damage caused by SARS-CoV2, although, to date this is not evidenced in clinical trials. (17) (NCT04364009, NCT04366232, NCT04362943, NCT04357366, NCT04324021, NCT04341584, NCT04362111, NCT04330638).

### Corticosteroids:

Corticosteroids have anti-inflammatory functions. The inhibition of excessive inflammation through the timely administration of glucocorticoids in the early stage of inflammatory cytokine storm effectively prevents the occurrence of ARDS (15). A recent retrospective study of 201 patients with COVID-19 in China found that, for those who developed ARDS, treatment with methylprednisolone was associated with a decreased risk of death (23/50 [46%] with steroids vs. 21/34 [62%] without; HR, 0.38 [95% CI,0.20-0.72]) (15). For patients with progressive deterioration of oxygenation indicators, rapid imaging progression or an excessive inflammatory response, the use of glucocorticoid in the short term (3-5 days) is appropriate, and the recommended dose is no more than the equivalent to methylprednisolone 1-2 mg/kg/day (15).

### Melatonin:

Melatonin (N-acetyl-5-methoxytryptamine) is used to treat sleep disorders, delirium, atherosclerosis, respiratory disease, and viral infections (4). It is not viricidal, but it has indirect anti-viral actions due to its anti-inflammation, anti-oxidation, and immune-enhancing features (4). In previous respiratory syncytial virus models, melatonin caused downregulation of acute lung oxidative injury, pro-inflammatory cytokine release, and inflammatory cell recruitment (4). Indeed, melatonin indirectly regulates ACE2 expression, a key entry receptor involved in viral infection of human coronavirus, including SARS-CoV-2 (6). A recent meta-analysis of a total of 22 randomized controlled trials suggested that a supplementary use of melatonin is associated with a significant reduction of TNF-α and IL-6 level (18). In another trial of patients who have severe multiple sclerosis, orally 25 mg/d of melatonin for six months promoted a significant reduction in serum concentrations of TNF-α, IL-6, IL-1β, and lipoperoxides (19). These findings support a rationale for melatonin use in viral diseases as a supplement, reducing the levels of circulating cytokines and pro-inflammatory cytokine levels in COVID-19 patients. Studies suggest that the use of melatonin is safe and well-tolerated (6). The adverse effects are limited to occasional dizziness, headache, nausea, and sleepiness (6). However, the direct evidence of melatonin application in COVID-19 is unclear. A clinical trial is ongoing to evaluate the efficacy of melatonin in the prophylaxis of COVID-19 among healthcare workers (NCT04353128).

### Miscellaneous agents or new lines of treatment to investigate?

Other drugs have demonstrated *in vitro* activity or have mechanisms purposed to inhibit SARS-CoV-2, including, baricitinib, imatinib, dasatinib, and cyclosporine (7). Current Chinese guidelines list interferons as an alternative for combination therapy (7). In one of the systematic reviews included, they report that interferon alone or in combination with ribavirin, lopinavir/ritonavir, has shown antiviral activity against coronavirus in studies extrapolated from SARS, Middle East respiratory syndrome coronavirus (MERS- -CoV) and some reports from COVID-19 (6).

Research efforts directed towards the design and development of vaccines for SARS--CoV-2 are increasing, and some related analyses are already being reported in distinct, parallel studies (20). Most COVID-19 vaccine private/industry developers are in North America followed by China, Asia (excluding China), Australia, and Europe. (21). Given the close genetic similarity between the structural proteins of SARS-CoV and SARS--CoV-2, immunological studies of the structural proteins of SARS-CoV could potentially aid the vaccine development for SARS-CoV-2. Focused specifically on the S and N proteins as these are known to induce potent and long-lived immune responses in SARS-CoV (20). About hyperimmune gammaglobulin and convalescent plasma from recovered patients can be a complementary therapy for COVID-19, but there is no evidence to recommend their use (7,15). Unfortunately, until now there is no current evidence to recommend any specific anti-COVID 19 treatment (7).

## CONCLUSIONS

Since the COVID-19 outbreak has begun, the global research and development effort in response to the COVID-19 pandemic to investigate potential therapies is unprecedented. Indeed, the volume and quick pace of published literature is continually changing. However, despite all the efforts done, no treatments have been shown effectiveness to date and randomized controlled clinical trials with high-quality evidence even in the middle of a pandemic are needed.
